# Bench, bed and beyond: Communication and responsibility in decentralised tuberculosis care

**DOI:** 10.4102/hsag.v24i0.1208

**Published:** 2019-09-30

**Authors:** Jennifer Watermeyer, Claire Penn, Megan Scott, Tshegofatso Seabi

**Affiliations:** 1 Health Communication Research Unit, School of Human and Community Development, University of the Witwatersrand, Johannesburg, South Africa

**Keywords:** Communication, Responsibility, Tuberculosis, Qualitative Research, Decentralised Care

## Abstract

**Background:**

South Africa faces one of the world’s worst drug-resistant tuberculosis epidemics. Implementing successful care in this context has proven challenging for a number of reasons. Communication is an essential yet neglected feature of care and research in the field of tuberculosis.

**Aim:**

The primary aim of this qualitative study was to explore communication facilitators and barriers at several tuberculosis care sites. In this article, we focus on communication practices across the chain of diagnosis, treatment, discharge and follow-up in decentralised care approaches and present evidence of gaps in communication.

**Setting:**

The study was conducted at three tuberculosis care sites in two South African provinces.

**Methods:**

Participants included healthcare workers, patients, community members and home-based carers. Data included 79 interviews, 4 video-recorded interactions between patients and healthcare workers, and ethnographic observations at each site. We analysed the data using thematic analysis and a qualitative sociolinguistic framework.

**Results:**

Communication in decentralised care contexts is complex because of multiple sites and role players. Responsibility for communication seems to be unduly placed on patients, treatment guidelines are not implemented consistently across sites and assumptions are made about the role of others in the chain. Patient and healthcare worker reports suggest confusion and frustration.

**Conclusion:**

Communication in the South African tuberculosis care context appears fragile and current mechanisms for detecting flaws in the care chain are not sensitive to communication issues. We make recommendations for strengthening home-based care resources, providing team training and focusing on communication processes in monitoring and evaluating systems.

## Introduction

South Africa has one of the world’s worst tuberculosis (TB) epidemics and a growing drug-resistant TB (DR-TB) epidemic exacerbated by the HIV burden. Implementing successful TB care has proven a major challenge in this context for various reasons, including the disease burden, poverty, geographical challenges, the need for service expansion and infrastructure support, and lengthy treatment duration (Saidi, Salie & Douglas 2017; Shah et al. 2017; Sinanovic et al. 2015). Although the necessary resources are available to achieve the goal of successful TB control programmes, what seems to be lacking is a coordinated strategy for their implementation (Ogunbanjo 2017). We would add that perhaps one of the most critical yet less frequently acknowledged components of TB treatment and care is communication.

Communication in healthcare settings happens at many levels, between patients and healthcare workers, as well as between healthcare workers, centres of care and the communities they serve (Poole & Real 2003; Storey et al. 2014). Communication can be thought of as the ‘cement that holds teams together’ (Propp et al. 2010) and it plays an essential role in promoting patient adherence to treatment and continuity of care (Penn, Watermeyer & Evans 2011; Miller & DiMatteo 2015). The World Health Organization’s guidelines for DR-TB management emphasise communication and coordination as vital between hospital and outpatient facilities (World Health Organization 2014). These processes need to be in place before, during and after discharge of a hospital patient to mitigate non-adherence and ensure adequate psychosocial support, while patients navigate the healthcare system and their own illness.

The potential for gaps in communication exists in any care system and communication may be made more challenging by language and cultural differences, poor literacy, patients’ lack of familiarity with treatment regimens and terminology, and illness, which may render a patient less able to recall and understand information (Penn et al. 2011). Several authors highlight how communication is not always optimal in TB treatment and care (Shringarpure et al. 2016; Skordis-Worrall, Hanson & Mills 2010). The complex, lengthy nature of treatment can make communication a challenge, resulting in delayed treatment initiation, patient loss to follow-up, poor feedback and reporting, communication breakdowns between clinics and community health workers, and inconsistent patient management across facilities (Blaya et al. 2014; Okeyo & Dowse 2016). In the context of decentralised care, and particularly DR-TB care, there are added layers of complexity and responsibility because of the involvement of multiple role players and centres of care. This approach requires smooth coordination and communication between each point of care in the chain of diagnosis, treatment and follow-up processes. Patients may start at one centre and get transferred to another centre or sent home into their community, making communication at these transition stages crucial for ensuring continuity of care.

Barriers to communication can occur at any of these points, but we would argue that they might be more difficult to detect and address once the care becomes decentralised. Not enough attention has been paid to the process and multidimensional nature of communication, the impact of contextual factors, and particularly the notion of responsibility for communication. Current national guidelines and suggestions for programme monitoring and evaluation do not include an explicit focus on communication and instead emphasise statistics and target reviews as measures of efficiency (Department of Health 2014).

The primary aim of this study was to explore communication facilitators and barriers identified at several TB care sites. In this article, we describe the critical role of communication across the chain of diagnosis, treatment and follow-up in decentralised TB care. We argue that current mechanisms for detecting flaws in the chain are probably not sensitive to communication issues. We suggest how qualitative methods can add evidence to this debate and highlight the direction that more efficient interventions and evaluations of process might assume.

## Methods

We chose an explorative qualitative design in line with previous research completed in the field of HIV. We conducted the study in two provinces of South Africa – Gauteng and Mpumalanga – at both centralised and decentralised TB care sites where we had established relationships.

### Setting

Gauteng province, the country’s urban economic hub, is culturally and linguistically diverse and home to many migrant workers. During 2015 and 2016, 49 661 TB and 1122 multidrug-resistant-tuberculosis (MDR-TB) cases were diagnosed in Gauteng. The TB cure rate was 87.1% (Gauteng Department of Health 2016). Our research sites in the province included two state hospitals: a centralised DR-TB treatment hospital (GP1) and a decentralised TB clinic within a secondary hospital (GP2). Mpumalanga is a rural province facing challenges such as unemployment, poverty, limited infrastructure, poor service delivery and geographical barriers. During 2013, 19 263 TB cases were reported in Mpumalanga province, of which 865 (4.5%) were MDR-TB cases. The TB cure rate was 67% (Musoke & Michel 2016). Our research site in this province was a rural decentralised HIV and TB primary healthcare clinic (MP).

### Data collection

We utilised a purposive sampling approach based on participants’ willingness and availability to participate. Healthcare worker, home-based care worker and patient participants were selected via purposive sampling and invited to participate in the study. Potential participants included all healthcare workers involved with TB care at each site, all home-based care workers associated with the MP clinic (note that home-based care workers were only interviewed at the MP site as there were none based at the GP sites at the time of the study), patients who had been diagnosed with TB and patients who were attending the sites during the data collection period. Patients who were too ill or not able to engage in an interview were not included. Community informants were selected via convenience sampling; specific inclusion criteria in terms of age and gender were not employed. Minors were not included in the study.

Linguistically matched and trained research assistants played a supportive and interpretive role at all sites. Interview guidelines were based on similar studies conducted in this context (Penn & Watermeyer 2018). A detailed summary of the data collected is provided in Table 1. Demographic details of the participants are included in Table 2.

**TABLE 1:** Data collection methods and participants across sites.

Data collection methods across participant groups	Centralised specialised hospital (GP1)	Decentralised secondary hospital (GP2)	Decentralised primary healthcare clinic (MP)
Interviews: Healthcare workers (HCWs) (*n* = 15)	5	2	8
Interviews: Patients (Ps) (*n* = 23)	10	4	9
Interviews: Home-based carers (HBCs) (*n* = 11)	-	-	11
Interviews: Community members (Cs) (*n* = 30)	-	15	15
Interactions: Nurses-Patients (*n* = 4)	-	4	-

**TABLE 2:** Demographic details of participants.

Participant groups	GP1 interviews	GP2 interviews	MP interviews	GP2 interactions
**Ps**				
*n*	10	4	9	4
***Gender***				
Female	5	2	6	2
Male	5	2	3	2
Age range	20 – 60 years	30 – 50 years	30 – 70 years	30 – 50 years
Language(s)	Sesotho, Afrikaans, isiZulu, isiXhosa, English	IsiZulu, English	SiSwati	IsiZulu, English
**HCWs**				
*n*	5	2	8	2
***Gender***				
Female	5	2	5	2
Male	-	-	3	-
Age range	30 – 60 years	30 – 50 years	20 – 55 years	30 – 50 years
Language(s)	IsiZulu, isiXhosa, Sesotho, Sepedi, English	IsiZulu, English	SiSwati, English	IsiZulu, English
Average years of experience	11.5 (range 1.5 – 24)	[Not available]	3.8 (range 1 – 7)	[Not available]
**HBCs**				
*n*	-	-	11	-
***Gender***				
Female	-	-	11	-
Male	-	-	-	-
Age range	-	-	20 – 65 years	-
Language(s)	-	-	SiSwati, English	-
**Cs**				-
*n*	-	15	15	-
***Gender***				
Female	-	7	11	-
Male	-	8	4	-
Age range	-	20 – 70 years	18 – 45 years	-
Language(s)	-	English, isiZulu, Afrikaans, Sesotho	Siswati, English	-

HCWs, healthcare workers; HBCs, home-based carers; Cs, community members.

Data included the following:

- Seventy-nine audio-recorded semi-structured interviews with healthcare workers (HCWs) (primarily nurses and counsellors), patients (P), lay community members (C) and home-based carers (HBC). We explored illness experiences, explanations of causation, treatment-seeking paths, communication issues and perceived challenges to TB care. The length of the interviews ranged from 8 to 15 min.
- Four video-recorded interactions involving discussions between nurses and patients about treatment, adherence and referrals. These took place in a private consultation room and lasted for 11 min on average. (Note that a limited sample of interactions was included from only one site, as the other sites did not grant permission for video recording and the nature of the sociolinguistic analysis warranted a small sample of interactional data.)
- Ethnographic observations of sites, informal staff discussions and community home visits, recorded via field notes.

### Trustworthiness

The project achieved trustworthiness in a number of ways. Credibility was achieved via triangulation of different types of data collected from different points of view across several sites, via the use of audio and video recording to document the data, and via a peer debrief process conducted during the analysis process. Transferability was achieved via purposive sampling of participants. Dependability was also achieved via triangulation, as well as through the first author’s running account of the research process of the project in a research journal. Confirmability was achieved via an audit trail that involved careful checking and rechecking of data sources, findings from analysis and process notes amongst members of the research team and with research assistants.

### Data analysis

All data were transcribed verbatim and translated into English where necessary. We conducted debrief sessions with the assistants to ensure reliability of translation. We analysed the interviews using thematic analysis (Braun & Clarke 2013). For the purpose of this article, we focused on themes related to communication, although other themes were identified in the data (associated with the lived experience of TB and contextual issues such as poverty and traditional beliefs) (Watermeyer & Penn 2019). Each author read and re-read the transcripts from each data set independently and identified themes and subthemes. All authors then met to discuss the themes from each data set and collate them into common major themes (see Table 3), thus ensuring rigour. The interactions were analysed using a sociolinguistic framework that focused on sequential interactional elements as well as facilitators and inhibitors to communication (Heath, Hindmarsh & Luff 2010; Sidnell & Stivers 2013). We examined verbal and non-verbal behaviours (including eye gaze, head nods, facial expressions and body language) of the patients and nurses. Findings from the interactions, interviews and ethnographic field notes were triangulated.

**TABLE 3:** Themes and subthemes related to communication.

Themes	Subthemes
Communication between healthcare workers and patients	Burden of ensuring patient understanding Communication style Relationship between patients and HCWs
Communication between centres of care	Lack of effective communication between centres Need for standardisation of care Patient burden of communication
Communication between the clinic and community	Community perception of health centres Role of the clinic Home-based carers

HCWs, healthcare workers.

### Ethical considerations

Ethical clearance was obtained from the University of the Witwatersrand Human Research Ethics Committee (Medical) (reference number: M120211) and permission was obtained from the research sites and the provincial Departments of Health. Participants were invited to participate in the study. Research assistants at each site explained the objectives of the study to participants in their language of choice and a participant information sheet was provided. Written consent was obtained from all the participants except in cases of illiteracy where verbal consent was obtained.

## Results

**FIGURE 1:**
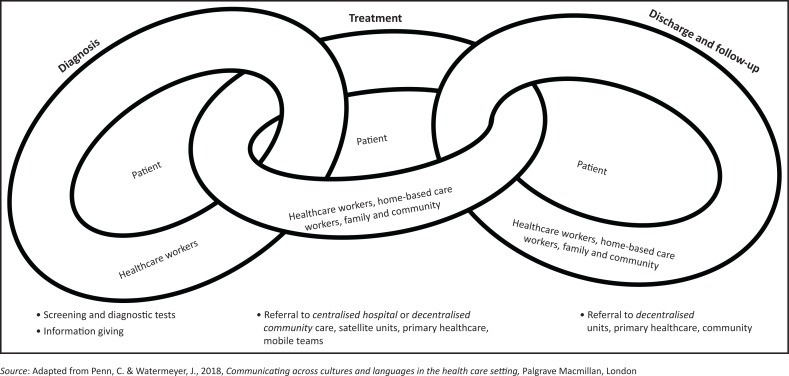
The chain of communication in TB care contexts.

We identified a chain of communication in TB care contexts encompassing activities involved in diagnosis, treatment, discharge and follow-up. This chain includes communication between healthcare workers and patients, healthcare worker to healthcare worker, across centres of care and between healthcare centres and the community. We have attempted to illustrate this concept in Figure 1. The phrase ‘bench, bed and beyond’, which has been used in relation to diagnostic TB testing (Dorman 2010), seems particularly encompassing of the idea of a chain of communication. We observed a wide variety of practices and perceptions across sites, with evidence suggesting that the links in the chain are not always effective.

We have chosen to present our results according to the themes and subthemes identified in the data set (see Table 3). We illustrate these themes with data excerpts.

### Communication between healthcare workers and patients

Although some patients felt supported by healthcare workers, others felt burdened by trying to remember all the information provided and did not feel able to request clarification:

‘She [*nurse*] should give me a paper to go home with. I will forget the information.’ (P2-GP2, female, 30 years old)

‘Sometimes you get scared to ask some questions.’ (P10-GP1, female, 35 years old)

A number of patients described negative experiences of diagnosis at other clinics and highlighted the lack of information provided about their diagnosis:

‘Where I was [at *the other clinic*] they did not tell me that I have TB.’ (P7-MP, female, 55 years old)

‘This clinic made me happy a lot because it diagnosed this sickness. … I started at [*another hospital*], taking bloods for 3 years. Still they were not finding the sickness … I then came and ended up going to [*another clinic*]. … It is far. I went and took bloods. I say that these clinics are not working, still not succeeding … it was an illness that could not be found. I even told my sister that it is better I die because there is nowhere I could go, special doctors I have been [*to*], still they are saying there is nothing, the bloods came saying I have nothing. How, because I can feel that I am sick. … It is then I came here, I took bloods, and [*it*] was found, the sickness was found.’ (P9-MP, male, 35 years old)

In the context of TB, and particularly DR-TB care, there is a large amount of information that needs to be conveyed to patients, particularly during the diagnosis and treatment processes. In the interactions we analysed, much time was spent on topics such as adherence strategies, healthy eating, the importance of avoiding traditional cleansing remedies, how to take HIV medication and maintaining safe sex practices.

We observed a tendency towards ‘information dumping’ by the nurses in these interactions, often with little patient participation other than head nods. For example, nurses spent much time explaining when medications should be taken. Some of the information conveyed was complex, for example around the process of decentralised care, how to take medications at home and the need to attend a local clinic to receive injections.

Despite spending considerable time on information giving, several healthcare workers indicated that they do not feel confident about patients’ understanding and potential to adhere:

‘They do not really know anything about TB.’ (HCW4-MP, female, 25 years old)

‘You give them treatment to take home, you’re not even sure that they are taking the treatment … but believing that they do.’ (HCW3-MP, female, 25 years old)

### Communication between centres of care

We observed limited communication between some care facilities. The burden is often placed on patients to explain to the referral clinic how they were managed at the previous health facility. Patients are required to act as a ‘middle man’ and much of the responsibility for ensuring continuity of care seems to be placed on the patient.

In several instances in our data set, we observed nurses telling patients how to instruct nurses at the next centre about adherence monitoring, injection tracking and recording of these tasks, as evidenced in Boxes 1 and 2 (bold added for emphasis).

BOX 1 Case 2.

**Table:** 

217	N:	Kukuwe ukuthi basebenza kanjani
		***How they work is entirely up to them***
218	P:	Ya
		*Yes*
219	N:	Fanele babone ukuthi uyaphuza. Ene ubatshele uSister N***
		*So they can see that you are taking them. **Tell them** sister*
220		uthe niyaticka la, niyakhombisa ukuthi uyaphuza lomuntu
		N*** says they must tick here, it will show that you are
221		amapilisi.
		*taking the pills.* ((points to document while she writes))
222	P:	Ya
		*Yes*

BOX 2 Case 4.

**Table:** 

115	N:	Then, abosister fanele bathicke la ((points to document))
		*Then **the sisters need to tick here***
116		ukuthi bayakwenzisa, nangapha bayaticka. Na la ((points to
		*to show that they are making you do it. Even here*
117		document))fanele baticke ukuthi bakhombise wena (.) And then
		***they need to tick** to show that you took them yourself* (.) *And then*
118		lana ((points to document)) lana fanele babhale ukuthi
		*here here **they need to write** how*
119		uprogressa kanjani, every month babhale ukuthi uprogressa
		*you’re progressing, every month they write how you are*
120		kanjani. ((points to document))
		*progressing.*
…		
124	N:	ubakhombise angithi?
		***You are going to show them** right?*
125	P:	Ee.
		*Yes.*
126	N:	Because angibazi abosister ukuthi bazobe bayenzani.
		***Because I don’t know what they will do.***

The nurses’ comments in Box 1 (line 217) and Box 2 (line 126) indicate that despite the availability of treatment guidelines, practices across sites are not standardised.

In contrast, however, healthcare workers at one of the sites (GP1) reported open communication between themselves and other centres after patients are discharged, particularly regarding patient discharge and follow-up:

‘Some patients they default, some they don’t come for check-ups. … We have to trace them [*and find out*] where are they, communicate with their clinics, when they are discharged we have to communicate … because when you discharge them here we take them to their nearest clinics so we have to communicate with the clinics if they are not following up the check-up dates.’ (HCW1-GP1, female, 35 years old)

These healthcare worker reports were further supported by feedback from some patients at the same site:

‘When I can see the medication is nearly finished … I call the sister, I just want to confirm that the medication [*is*] there at the clinic, if there is no medication, then the sister just call here [t*his hospital*]. They just ask them, where is my medication? And then if the medication is coming early, arrive early, the sister call me come and collect your medication.’ (P1-GP1, male, 55 years old)

We also noted several instances where healthcare workers made reference to links in the chain of care, either to what had occurred at a previous point of care or to what was still to come. For example, in the quote below, the healthcare worker highlights the importance of documenting care processes for the next point of care:

‘When you do something you have to note down so that the next person must be updated of what is happening.’ (HCW3-MP, female, 25 years old)

### Communication between clinic and community

Some participants spoke about the links between the community and the clinic. At the rural Mpumalanga site, healthcare workers discussed the importance of providing education to the community and initiating links with community members. Similarly, community members felt a responsibility to link with the clinic:

‘Bring the clinic to the people.’ (HCW1-MP, female, 35 years old)

‘We must come to the clinic because they [*staff*] will not come to the community and get us.’ (C1-MP, male, 30 years old)

‘We should be working with them [*the doctors*].’ (C9-MP, male, 30 years old)

Community members at the Gauteng site spoke about the importance of clinics in promoting education about TB that is accessible to communities:

‘I think it’s much better for a person to explain it [*TB*] and the situation than what you read. You know some people can read but then sometimes there are words that you don’t understand. So I’m not going to take my dictionary out so that I can understand that word. So what do I do? I just blablabla over it and it’s finished. But if you have someone who understands it and explains it to you, then you understand much better what it’s all about. Because in your explaining you are going to teach me how to assist the next person as well.’ (C13-GP2, female, 55 years old)

Perhaps the most crucial link in the chain of communication between community and clinic is the home-based care worker. Home-based carers at the Mpumalanga site described their role as including tasks such as encouraging patients to go to the clinic, collecting sputum, providing physical and home activity-assisted care to patients who live alone and educating communities about contagion. The carers play a particularly important role in promoting adherence:

‘We go to the community and when we find patients that have TB, we make them aware, ask how are they drinking their treatment and check their [*diary*] cards and when we find that they say “this one is not drinking them well”, we sit down and talk with him/her and ask “why are you not drinking them well?” and [*they*] will say “there was a problem here and here and there”, you see.’ (HBC10-MP, female, 40 years old)

‘The patient must hear from someone who is talking from experience.’ (HBC3-MP, female, 40 years old)

The link between home-based carers and local decentralised clinics, however, is often not an explicit or officially recognised one:

‘We have been working for years and we have never received a stipend. Are we going to get it or not, we still don’t know … we also told the clinic matron and she does give us work … but we don’t get any benefit.’ (HBC6-MP, female, 30 years old)

Several home-based carers indicated that there are limited opportunities for communication with the healthcare workers at the local clinic about specific patient issues and they do not feel part of the healthcare team. They indicated their desire to be trusted by local clinics and included in discussions about community and patient-related issues:

‘I would like the staff to understand how much [*burden*] I carry outside there. There are people [*who*] believe that I play outside, I just go around the community doing nothing … If I am coming back from the community [*to*] the clinic, when reporting about a patient they [*staff*] must take it seriously… like there is Sister [*name*] who deals with TB, she is very supportive; every time when I come here she always pays attention, listens to what I am saying and also guides me. How can they support me [*further*]? If I have a professional nurse once a week and know that this day of the week I go out with her/him and be able to see the difficult cases and be there and again guide me.’ (HBC3-MP, female, 40 years old)

‘I am important just like them [*doctors*].’ (HBC7-MP, female, 45 years old)

## Discussion

Our study suggests a lack of uniformity between centres in the way that care is provided and guidelines implemented, as well as limited communication between centres and a tendency to work in silos rather than in a coordinated manner. As a result, a significant burden of communication is placed on patients and healthcare workers in terms of the volume of information to convey and the responsibility of ensuring continuity of care. This confirms the observations of some researchers (Churchyard et al. 2014) that the implementation of guidelines across care sites is problematic and inconsistent.

In particular, there is increased responsibility on patients to understand the complexities of the care approach and treatment regimen and communicate information to other care centres. In the context of poor literacy, poverty, linguistic diversity and geographic distance, this is not necessarily the right approach (Farmer 2000). A treatment diary card alone is not enough to ensure good communication across sites, and the primary responsibility for such communication should not be placed on the patient. In so doing, there is a strong likelihood of miscommunication and misunderstanding occurring, which may have deleterious effects on patient adherence to treatment regimens.

Prior research has demonstrated that in order to obtain positive patient outcomes, effective communication and coordination is not only necessary at the level of communication between healthcare workers and patients, but also across multidisciplinary teams and contexts (Poole & Real 2003; Portwig & Couper 2006). Our results show that current mechanisms of communication in TB care are often not sensitive to what matters to patients and healthcare workers. Healthcare workers seem to be making assumptions about the role of other healthcare workers and patients in the chain, and checks and balances are not in place.

## Recommendations

Although there are many challenges for implementing decentralised care, we believe that the links in the chain of communication in TB care can be strengthened. Just as patients need to feel empowered to take charge of their own health and treatment, so healthcare workers need to be empowered to take responsibility and initiate open lines of communication within their own teams, with other treatment centres and with communities. Focusing on improving and standardising organisational routines and practices, and particularly communication routines within and across sites, has the potential to alleviate patient and healthcare worker anxiety, enable patients to know where to go and what to do, and promote coordinated efficiency within and between sites of care (Miller & Apker 2002).

Effective communication is a critical component of patient-centred care. It necessitates sensitivity to patient needs, values and preferences, and the development of a partnership and a sense of joint responsibility between healthcare workers and patients (Schiavo 2013). Our study has highlighted a number of problems related to how healthcare workers give information to patients and how this information is understood. In line with a patient-centred approach to communication, there are several actions that healthcare workers can do to improve practices in this regard. Strategies to enhance information giving may include, for example, focusing on what information is most important for each consultation, breaking up information giving over multiple appointments, supplementing verbal information giving with written information, exploring the patient’s expectations and agenda for the consultation, and linking information giving to these aspects. We have written elsewhere about effective tools to verify patient understanding of information and identify misunderstandings (Watermeyer & Penn 2009), the most important of which is to elicit a demonstration of understanding by asking the patient to repeat back what they have been told.

There is a need for monitoring and evaluation systems that consider communication factors and contextual issues. Too often the guidelines focus in a task-oriented manner on statistics and patient records as measures of effective care (Lewin & Green 2009) rather than on examining process factors, such as communication and coordination within and across sites. A closer check of such factors using qualitative methods, as we have demonstrated in this article, is likely to increase the successful implementation of decentralised care approaches.

A communication specialist could play an important role in identifying potential barriers to communication and initiating opportunities across sites of care and with relevant role-players. Our experience has shown that communication skills training may encourage more efficient communication across the chain and assist healthcare workers to become more sensitive to potential points of breakdown and generate strategies for repair (Penn et al. 2011). Importantly, such training needs to focus on the entire care team (healthcare workers and mid-level workers such as home-based carers), rather than targeting specific professional groups (Penn & Watermeyer 2018).

Our findings on home-based carers playing an important yet undervalued role echo previous research in other contexts (Cataldo et al. 2015). They operate at the critical yet fragile interface of the medical and social worlds. Their key role as communication gatekeepers between healthcare workers, patients and the community needs to be further enhanced, valued and remunerated. Our study emphasises the need for clinics and communities to forge stronger connections in order to fight TB and promote adherence to TB treatment, and home-based carers have the potential to facilitate this link.

## Conclusion

Although this was an exploratory study based on a small group of participants at select sites across two provinces, the results have implications for other contexts where decentralised TB care models are implemented. This study addresses an important gap in existing literature and demonstrates the need for more research on communication practices in TB care contexts. Our findings confirm the importance of understanding the perspectives of different role players in a healthcare system as a starting point for developing locally attuned solutions. Furthermore, it shows the value of qualitative methods in highlighting the voices of the patient, healthcare worker and community, as well as revealing some of the challenges of communication within the multiple links in the chain of TB care systems.

The current TB care system appears fragile, particularly when it comes to communication. The chain of decentralised care involves many role players and centres that are expected to provide care but are not necessarily equipped to deal with the challenges posed by this model. If communication breaks down at one point, there may be negative implications further down the chain and potentially serious consequences for patient adherence. Continuity of care emerges when communication between centres, systems and individuals is coordinated. Without effective communication, the whole system is vulnerable to breakdown. This study provides some evidence for these breakdowns.

If decentralised TB care approaches are to be implemented successfully, then multiple dimensions of care must be considered and the critical role of communication should be foregrounded. There is a need to focus on communication at each level of TB care and develop specific guidelines to enhance communication, as has been done to some extent in HIV care (Kawonga, Blaauw & Fonn 2015; Penn & Watermeyer 2018). Health systems need to acknowledge and create supportive channels to address the demands of effective communication placed on healthcare teams and, by extension, on patients. This is particularly pertinent for decentralised care models that exist within contextually challenging settings such as South Africa.

## References

[CIT0001] Blaya J.A., Shin S.S., Yagui M., Contreras C., Cegielski P., Yale G. et al., 2014, ‘Reducing communication delays and improving quality of care with a tuberculosis laboratory information system in resource poor environments: A cluster randomized controlled trial’, *PLoS One* 9(4), e90110 10.1371/journal.pone.0090110 24721980 PMC3982951

[CIT0002] Braun V. & Clarke V., 2013, *Successful qualitative research: A practical guide for beginners*, Sage, London.

[CIT0003] Cataldo F., Kielmann K., Kielmann T., Mburu G. & Musheke M., 2015, ‘Deep down in their heart, they wish they could be given some incentives’: A qualitative study on the changing roles and relations of care among home-based caregivers in Zambia’, *BMC Health Services Research* 15(1), 36 10.1186/s12913-015-0685-7 25627203 PMC4324023

[CIT0004] Churchyard G.J., Mametja L.D., Mvusi L., Ndjeka N., Hesseling A.C., Reid A. et al., 2014, ‘Tuberculosis control in South Africa: Successes, challenges and recommendations’, *South African Medical Journal* 104(3), 234–248. 10.7196/SAMJ.7689 24893501

[CIT0005] Department of Health, 2014, *National TB management guidelines 2014*, viewed 25 May 2018, from https://www.health-e.org.za/wp-content/uploads/2014/06/NTCP_Adult_TB-Guidelines-27.5.2014.pdf.

[CIT0006] Dorman S.E., 2010, ‘New diagnostic tests for tuberculosis: Bench, bedside, and beyond’, *Clinical Infectious Diseases* 50(Supplement 3), S173–S177. 10.1086/651488 20397945

[CIT0007] Farmer P.E., 2000, ‘The consumption of the poor: Tuberculosis in the 21st century’, *Ethnography* 1(2), 183–216. 10.1177/14661380022230732

[CIT0008] Gauteng Department of Health, 2016, *Annual Report 2015–2016. Gauteng Department of Health*, viewed 25 May 2018, from http://www.gauteng.gov.za/government/departments/health/Annual%20Report/GDH%20Annual%20Report%202015-2016.pdf.

[CIT0009] Heath C., Hindmarsh J. & Luff P., 2010, *Video in qualitative research*, Sage, London.

[CIT0010] Kawonga M., Blaauw D. & Fonn S., 2015, ‘Exploring the use of social network analysis to measure communication between disease programme and district managers at sub-national level in South Africa’, *Social Science & Medicine* 30(135), 1–4. 10.1016/j.socscimed.2015.04.024 25931377

[CIT0011] Lewin S. & Green J., 2009, ‘Ritual and the organisation of care in primary care clinics in Cape Town, South Africa’ *Social Science & Medicine* 68(8), 1464–1471. 10.1016/j.socscimed.2009.02.013 19278764

[CIT0012] Miller K.I. & Apker J., 2002, ‘On the front lines of managed care: Professional changes and communicative dilemmas of hospital nurses’, *Nursing Outlook* 1, 50(4), 154–159. 10.1067/mno.2002.126111 12189350

[CIT0013] Miller T.A. & DiMatteo M., 2015, ‘Treatment adherence/compliance’, *The Encyclopedia of Clinical Psychology*, Wiley, Chichester.

[CIT0014] Musoke J. & Michel A.L., 2016, ‘Characteristics of tuberculosis patients and the evaluation of compliance to the national TB management guidelines at clinics in a rural community from Mpumalanga province, South Africa’, *Southern African Journal of Infectious Diseases* 31(4), 135–137. 10.1080/23120053.2016.1156879

[CIT0015] Ogunbanjo G.A., 2017, ‘Multi-drug-resistant tuberculosis (MDR-TB): Current situation in South Africa’, *South African Family Practice* 59(2), 5.

[CIT0016] Okeyo I. & Dowse R., 2016, ‘Community care worker perceptions of their roles in tuberculosis care and their information needs’, *Health SA Gesondheid* 21, 245–252. 10.1016/j.hsag.2016.05.004

[CIT0017] Penn C. & Watermeyer J., 2018, *Communicating across cultures and languages in the health care setting*, Palgrave Macmillan, London.

[CIT0018] Penn C., Watermeyer J. & Evans M., 2011, ‘Why don’t patients take their drugs? The role of communication, context and culture in patient adherence and the work of the pharmacist in HIV/AIDS’, *Patient Education and Counseling* 83(3), 310–318. 10.1016/j.pec.2011.02.018 21474263

[CIT0019] Poole M.S. & Real K., 2003, ‘Groups and teams in health care: Communication and effectiveness’, *Handbook of health communication*, Lawrence Erlbaum, Mahwah, NJ.

[CIT0020] Portwig G.H. & Couper I.D., 2006, ‘A qualitative study of the reasons why PTB patients at clinics in the Wellington area stop their treatment’, *South African Family Practice* 1, 48(9), 17-c 10.1080/20786204.2006.10873459

[CIT0021] Propp K.M., Apker J., Zabava Ford W.S., Wallace N., Serbenski M. & Hofmeister N., 2010, ‘Meeting the complex needs of the health care team: Identification of nurse-team communication practices perceived to enhance patient outcomes’, *Qualitative Health Research* 20(1), 15–28. 10.1177/1049732309355289 20019348

[CIT0022] Saidi T., Salie F. & Douglas T.S., 2017, ‘Towards understanding the drivers of policy change: A case study of infection control policies for multi-drug resistant tuberculosis in South Africa’, *Health Research Policy and Systems* 15(1), 41 https://doi.org/10.1186%2Fs12961-017-0203-y 28558838 10.1186/s12961-017-0203-y PMC5450238

[CIT0023] Schiavo R., 2013, *Health communication: From theory to practice*, John Wiley & Sons, Hoboken, NJ.

[CIT0024] Shah N.S., Auld S.C., Brust J.C., Mathema B., Ismail N., Moodley P., et al., 2017, ‘Transmission of extensively drug-resistant tuberculosis in South Africa’, *New England Journal of Medicine* 376(3), 243–253. 10.1056/NEJMoa1604544 28099825 PMC5330208

[CIT0025] Shringarpure K.S., Isaakidis P., Sagili K.D., Baxi R.K., Das M. & Daftary A., 2016, ‘“When treatment is more challenging than the disease”: A qualitative study of MDR-TB patient retention’, *PLoS One* 11(3), e0150849 10.1371/journal.pone.0150849 26959366 PMC4784928

[CIT0026] Sidnell J. & Stivers T., 2013, *The handbook of conversation analysis*, Wiley, Chichester.

[CIT0027] Sinanovic E., Ramma L., Vassall A., Azevedo V., Wilkinson L., Ndjeka N. et al., 2015, ‘Impact of reduced hospitalisation on the cost of treatment for drug-resistant tuberculosis in South Africa’, *International Journal of Tuberculosis and Lung Disease* 19(2), 172–178. 10.5588/ijtld.14.0421 25574915 PMC4447891

[CIT0028] Skordis-Worrall J., Hanson K. & Mills A., 2010, ‘Confusion, caring and tuberculosis diagnostic delay in Cape Town, South Africa’, *International Journal of Tuberculosis and Lung Disease* 14(2), 171–180. 20074408

[CIT0029] Storey D., Seifert-Ahanda K., Andaluz A., Tsoi B., Matsuki J. & Cutler B., 2014, ‘What is health communication and how does it affect the HIV/AIDS continuum of care? A brief primer and case study from New York City’, *Journal of Acquired Immune Deficiency Syndromes* 66, S241–S249. 10.1097/QAI.0000000000000243 25007193

[CIT0030] Watermeyer J. & Penn C., 2009, ‘“Tell me so I know you understand”: Pharmacists’ verification of patients’ comprehension of antiretroviral dosage instructions in a cross-cultural context’, *Patient Education and Counseling* 75, 205–213. 10.1016/j.pec.2008.09.009 19070986

[CIT0031] Watermeyer J. & Penn C., 2019, ‘Community perspectives on tuberculosis care in rural South Africa’, *Health and Social Care in the Community* 27, 182–190. 10.1111/hsc.12637 30159955

[CIT0032] World Health Organization, 2014, *Companion handbook to the WHO guidelines for the programmatic management of drug-resistant tuberculosis*, viewed 25 May 2018, from http://apps.who.int/iris/bitstream/10665/130918/1/9789241548809_eng.pdf?ua=1&ua=1. 25320836

